# Fiber Mediated Receptor Masking in Non-Infected Bystander Cells Restricts Adenovirus Cell Killing Effect but Promotes Adenovirus Host Co-Existence

**DOI:** 10.1371/journal.pone.0008484

**Published:** 2009-12-29

**Authors:** Johan Rebetz, Manli Na, Changqing Su, Bo Holmqvist, Anna Edqvist, Cecilia Nyberg, Bengt Widegren, Leif G. Salford, Hans Olov Sjögren, Niklas Arnberg, Qijun Qian, Xiaolong Fan

**Affiliations:** 1 The Rausing Lab, Lund University, Lund, Sweden; 2 Department of Cell and Organism Biology Lund University, Lund, Sweden; 3 Department of Oncology and LBIC Optical Imaging Unit, Lund University, Lund, Sweden; 4 Division of Virology, Department of Clinical Microbiology, Umeå University, Umeå, Sweden; 5 Laboratory of Gene and Viral Therapy, Eastern Hepatobiliary Surgical Hospital, the Second Military Medical University, Shanghai, China; 6 Department of Physiology, Life Science College, Beijing Normal University, Beijing, China; Karolinska Institutet, Sweden

## Abstract

The basic concept of conditionally replicating adenoviruses (CRAD) as oncolytic agents is that progenies generated from each round of infection will disperse, infect and kill new cancer cells. However, CRAD has only inhibited, but not eradicated tumor growth in xenograft tumor therapy, and CRAD therapy has had only marginal clinical benefit to cancer patients. Here, we found that CRAD propagation and cancer cell survival co-existed for long periods of time when infection was initiated at low multiplicity of infection (MOI), and cancer cell killing was inefficient and slow compared to the assumed cell killing effect upon infection at high MOI. Excessive production of fiber molecules from initial CRAD infection of only 1 to 2% cancer cells and their release prior to the viral particle itself caused a tropism-specific receptor masking in both infected and non-infected bystander cells. Consequently, the non-infected bystander cells were inefficiently bound and infected by CRAD progenies. Further, fiber overproduction with concomitant restriction of adenovirus spread was observed in xenograft cancer therapy models. Besides the CAR-binding Ad4, Ad5, and Ad37, infection with CD46-binding Ad35 and Ad11 also caused receptor masking. Fiber overproduction and its resulting receptor masking thus play a key role in limiting CRAD functionality, but potentially promote adenovirus and host cell co-existence. These findings also give important clues for understanding mechanisms underlying the natural infection course of various adenoviruses.

## Introduction

Adenovirus infections are endemic in all human populations regardless the quality of their health standards. Although adenovirus infections can be persistent or latent, they are mostly acute or self-limiting [1], [2]. As acute adenovirus infection results in cell lysis, serotype 5 adenovirus (Ad5) based conditionally replicating adenoviruses (CRAD) have been developed as oncolytic agents [3]. The CRADs have been engineered either by controlling E1A expression via cancer cell specific promoters, or by deletion of adenoviral gene functions essential for viral replication in normal cells but not in tumor cells [3]. A large number of cell culture and xenograft tumor model studies have shown the potential power of CRAD in cancer therapy. However, successful translation of these promising pre-clinical results to the benefit of cancer patients remains elusive [4].

Various strategies have been applied to improve the cancer cell killing capacity of CRAD. For example, more stringent cancer cell specific promoters have been utilized to control E1A expression for improved specificity of CRAD replication in cancer cells [5]–[7]. Binding of adenovirus fiber proteins to host cell receptors is the very first step in initiating adenoviral infection in many cell types, and critically determines whether a given cell type is permissive to adenovirus infection. So by fiber re-targeting, novel tropism has been engineered in CRAD, enabling CRAD to infect different/multiple types of cancer cells [8], [9]. However, a key challenge in utilizing CRADs as cancer therapy agents appears to be their inefficient spreading capacity which limits infection propagation [10], [11]. Also, as most individuals have neutralizing antibodies against Ad5, CRAD can potentially be cleared following intra-tumoral application [12], [13]. But even in the absence of an antiviral immune response, adenoviruses failed to eradicate established xenograft tumors, despite ongoing viral replication [10], [11]. Spatial constraints were suggested as the major reason for this dilemma but there are still no satisfactory explanations for the lack of clinical success with oncolytic adenovirus.

The production of fiber protein molecules in great excess to the actual need for adenoviral particle assembly has been detected in the life cycle of several adenovirus serotypes [14]–[16]. The function of excessive fiber production is unclear, but it has been suggested to be important for efficient adenoviral particle assembly [15]. Another role for the fiber can be in propagation of infection. Ad5 fiber molecules can disrupt CAR-mediated cell-cell adhesion between airway epithelial cells thereby facilitating and increasing adenovirus spread across epithelial cell layers, which led to the hypothesis that an important function of excessively produced fiber molecules was to increase progeny adenovirus spread from infected cells to non-infected bystander cells [17]. We have investigated the role of fiber overproduction in the course of multiple rounds of CRAD or wild type (WT) adenovirus infection. We show that fiber overproduction in the few initially infected cells and their secretion prior to CRAD release results in receptor masking in the bulk of non-infected neighboring cells, thereby limiting infection efficiency of progeny CRAD viruses. This process represents a key detrimental feature limiting CRAD cancer cell killing efficiency. We observed the fiber overproduction and its resulting receptor masking during infection by both CAR- and CD46-binding WT adenoviruses, suggesting that the fiber overproduction and the tropism-specific receptor masking critically controls adenovirus propagation and persistency of infection.

## Results

### Cancer Cell Killing Effect of CRAD Infection at Low MOI

**Figure 1:**
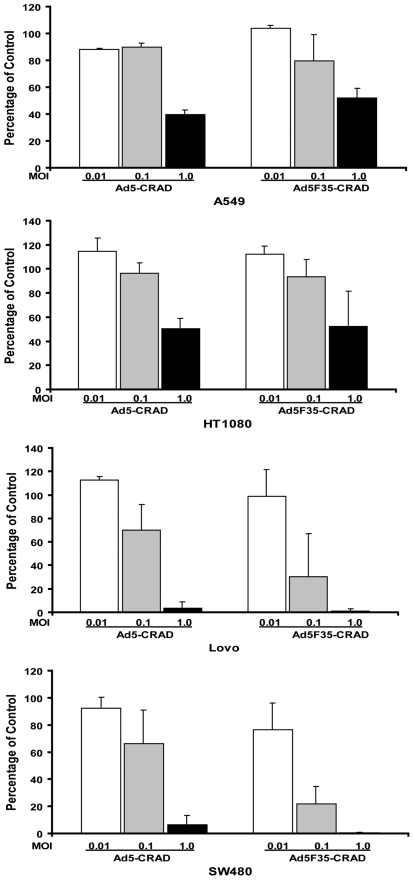
Cancer cell killing of CRAD infection at low MOI. Mean ± SD (n = 3) of total cell numbers of CRAD infected cell cultures relative to non-infected control cultures at one-week post infection are shown.

Assuming that CRADs have an exponential propagation capacity, any permissive cancer cell culture should show an exponential increase in infected cells following initial round of CRAD infect-release-reinfect cycles, even when the infection is initiated in a few cells. To simulate such hypothetical conditions, we used two CRADs, Ad5-hTERT-E1A-GFP and Ad5F35-hTERT-E1A (hereafter referred to as Ad5-CRAD and Ad5F35-CRAD, respectively), to infect cancer cells at very low MOI. In these two CRADs, the expression of E1A is controlled by the *human telomerase reverse transcriptase* (*hTERT*) promoter, which is preferentially active in most tumor cells [5]. When adenocarcinoma lung cancer A549 cells were infected with Ad5-CRAD or Ad5F35-CRAD at MOIs of 0.01, 0.1 and 1.0, a significant reduction in cell numbers at one week post infection was only observed in infections at a MOI of 1.0, but not at 0.1 or 0.01 (Figure 1). However, the latter cultures were destroyed in the third or fourth week post infection, demonstrating very slow kinetics of cell killing. Similar findings were observed in lung cancer HT1080, and colon cancer Lovo, SW480 cells (Figure 1). These CRADs indeed propagate (see data below) and super-infection with these CRADs at a MOI of 10 resulted in complete killing of A549 cell culture after 3 days. These data indicate that the spread of CRAD infection is hindered by a hitherto unknown mechanism, and this mechanism prolongs the co-existence between CRAD propagation and cell survival in culture.

### Tropism-Specific Decrease in Receptor Detectibility in Infected and Non-Infected Cells

**Figure 2:**
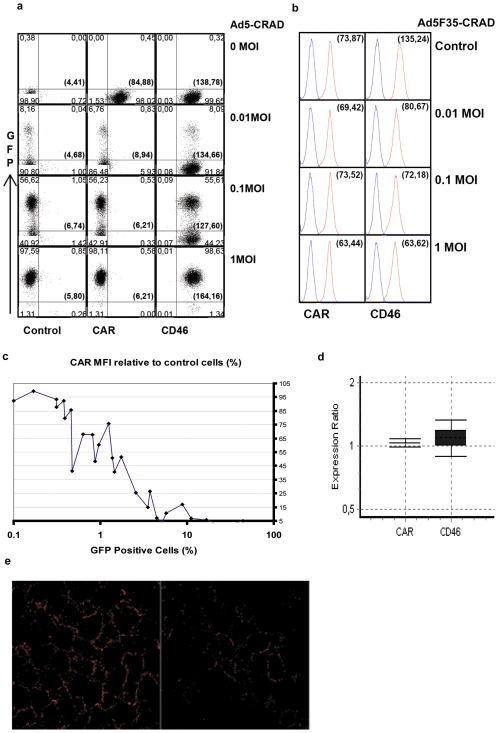
Decrease of receptor detectibility following CRAD replication in a small fraction of cells. (A) GFP expression and CAR, CD46 intensity in A549 cells at one week following Ad5-CRAD infection at indicated MOI. Numbers in each quadrate represent the phenotypic distribution of the cells according to GFP expression and CD46 or CAR mAb staining intensity. The numbers in brackets represent the mean fluorescence intensity (MFI) of CAR or CD46 staining of all cells. Data are representative for (C). (B) CAR and CD46 intensity in A549 cells at one week following Ad5F35-CRAD infection. The numbers in brackets represent the MFI of CAR or CD46 staining (red line in each histogram) of all cells. The blue lines represent isostype control antibody staining. (C) Inverse correlation between CAR intensity and the percentages of GFP^+^ cells following Ad5-CRAD infection at low MOIs. Each dot represents one independent assay for GFP expression and CAR MFI relative to the control non-infected cells. (D) The ratios of CAR or CD46 mRNA expression level (filled box, mean ± SD) and its 95% confidence intervals between Ad5-CRAD infected (with ∼2% GFP^+^ cells) and non-infected A549 cultures. (E) Representative CLSM analyses of RcmB binding in A549 control culture (left panel) or cultures at day 6 post infection with Ad5-CRAD at a MOI of 1.0 with ∼1.5% cells being GFP^+^ (right panel).

The initiation of adenoviral infection in most cell types is critically dependent on binding of adenoviral particle fiber to host cell receptors. Ad5-CRAD binds CAR as a cellular receptor [18], whereas Ad5F35-CRAD contains the knob and shaft domains of Ad35 fiber gene and thus utilizes CD46 as a receptor [9]. Of note, the GFP cDNA is engineered into the E1B locus in Ad5-CRAD [6], such that CRAD replication can be directly traced in living cells via GFP expression. Using flow cytometry analysis, we assessed CAR and CD46 intensity in A549 cultures following infection with Ad5-CRAD or Ad5F35-CRAD vector at low MOI. At one week post infection with Ad5-CRAD, CAR was undetectable in both GFP^+^ cells (with viral replication) and GFP^-^ cells (without viral replication) as assessed with RmcB mAb (Figure 2A). In contrast, no measurable decrease of CD46 intensity was observed as assessed with E4.3 anti-CD46 mAb. Conversely, a decrease of up to 50% in CD46 intensity, but no obvious effect on CAR intensity, was observed in cultures following infection with Ad5F35-CRAD (Figure 2B). Thus, the decrease in receptor intensity is tropism-specific, and this is unlikely a consequence of global inhibition of gene expression due to adenovirus infection. Following Ad5-CRAD infection, an inverse correlation between the percentages of GFP^+^ cells and the relative CAR intensity was observed (Figure 2C). Importantly, Ad5-CRAD replication in only 1% of the cells resulted in a ∼50% decrease of CAR intensity in all cells, and a further decrease close to the background level was observed when 10% of the cells were infected (Figures 2A and 2C). And, no decrease in CAR expression, as assessed by CAR mRNA copy numbers, was detected in Ad5-CRAD infected A549 cultures (with ∼2% GFP^+^ cells) compared to non-infected cultures (Figure 2D). Furthermore, an analogous dose-dependent, tropism-specific decrease of CAR and CD46 intensity was detected in A549 cells following infection with WT Ad5 and Ad11, respectively (see below). We performed confocal laser scanning microscopy (CLSM) analysis to visualize RmcB mAb binding to individual cells in Ad5-CRAD infected culture. Although a quantitative measurement was not feasible, compared to non-infected cultures (Figure 2E, left), a relatively weaker RmcB binding was generally appreciated in Ad5-CRAD infected cultures (Figure 2E, right). These findings together demonstrate that CRAD infection of a small population of cancer cells resulted in diminished receptor intensity in both infected and non-infected bystander cells in a tropism specific manner.

### Excessive Fiber Molecule Production from the First Round of CRAD Infected Cells Mask Adenoviral Receptors

**Figure 3:**
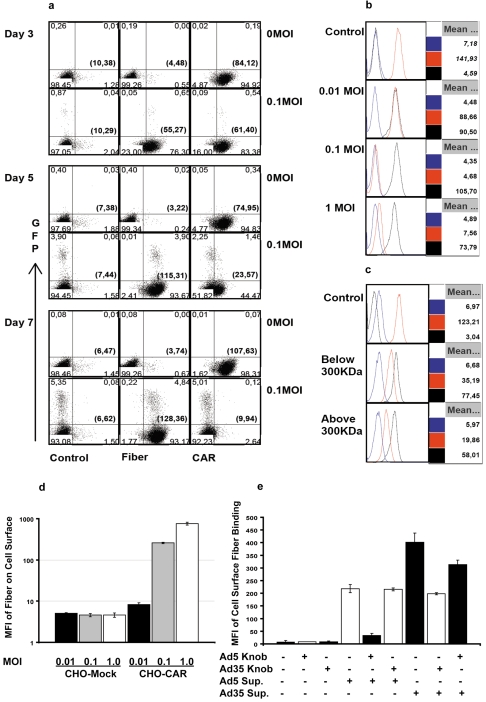
Fiber binding and receptor masking in infected and non-infected cells. (A) Progressive fiber binding and concomitant decrease in CAR intensity in both infected and non-infected A549 cells were similar following infection with Ad5-CRAD at low MOI. Representative data from 3 independent studies are shown. (B) Supernatants from Ad5-CRAD infected A549 cultures conferred cell surface fiber binding and concomitant decrease in CAR intensity in non-infected A549 cells. Non-infected cells were incubated for 2 hr with A549 culture supernatants harvested at one week post infection with Ad5-CRAD at the indicated MOI, washed and analyzed for CAR and fiber intensity. Data shown are representative histograms of 3 experiments performed at 37°C. The MFIs of isotype control (blue lines), CAR (red lines) and fiber (black lines) staining of all cells are indicated at the right part of each histogram. (C) Free fiber molecules in <300 KDa supernatant fraction from Ad5-CRAD infected A549 cultures conferred the same effect as in (B). Supernatants used in (B) were centrifuged at 108 000 g for 1 hr, fractioned through a membrane with 300 KDa cut-off, and subsequently used in binding experiments as in (B). (D) Supernatants from A549 cell cultures at one week following infection with Ad5-CRAD at the indicated MOIs conferred fiber binding only to CAR expressing CHO cells. The mean ± SD (n = 2) of fiber binding MFI are shown. (E) Supernatant fiber binding to fresh A549 cells was inhibited by recombinant Ad5 or Ad35 fiber knob molecules in a tropism specific manner. The mean ± SD (n = 2) of fiber binding MFI to A549 cells are shown.

To investigate the mechanisms of diminished receptor intensity, we tested the hypothesis that the initial round of CRAD infection in few cancer cells produced excessive amounts of free fiber molecules, which bound to receptors in both infected and non-infected cells, and thereby masked these receptors. Using 4D2 mAb, previously reported to recognize fiber tail domain of Ad2 and Ad5 [15], [19], we studied the effect of fiber production on receptor intensity in the course of CRAD infection. Following infection of A549 cell cultures with Ad5-CRAD at a MOI of 0.1, we observed a progressive increase of fiber binding to GFP^+^ as well as GFP^−^ cells from day 3 to 7 post infection (Figure 3A). By day 3 post infection, 76% of the cells had bound fibers whereas only <1% of the cells were infected by CRAD; by day 5, the binding had increased to 94% whereas only <4% of the cells being infected. Similar findings were also observed when the anti-fiber mAb clone 2A6, which recognizes the N-terminal half of trimeric fiber shaft domain was used (data not shown). Concomitantly, a progressive decrease in CAR intensity (from 72% of the control on day 3, to 31% on day 5, to non-detectable on day 7), but not of CD46 was seen (Figure 3A). Similar trends of increasing fiber binding and decreasing CAR intensity were observed in HT1080, Lovo, SW480, and breast cancer SK-Br-3 cells following Ad5-CRAD infection (Figure S1).

Excessive fiber molecules were also detected in the supernatant of CRAD infected cultures. High intensity fiber binding with concomitant decrease of CAR intensity was observed when A549 cells were incubated with the supernatants from Ad5-CRAD infected cultures, irrespective of whether the experiments were performed at 37°C (permissive for receptor mediated internalization) (Figure 3B) or at 0°C (non-permissive for receptor mediated internalization) (data not shown).

Supernatants from Ad5-CRAD infected cultures can contain free fiber molecules, as well as large amounts of defective viral particles and penton complexes with intact fiber. To substantiate the binding of free fiber molecules to receptors, a supernatant fraction containing free trimeric fiber molecules (∼200 kilodalton (KDa)) was prepared by depletion of viral particles with centrifugation at 108 000 g for 1 hr followed by filteration through a membrane with cut-off size 300 KDa. The free fiber-containing fraction (<300 KDa fraction) was found to confer a high intensity fiber binding with a concomitant decrease in CAR detectability in fresh A549 cells (Figure 3C). The same findings were also observed with the >300 KDa fraction, which besides free fiber also likely contained viral particle non-associated penton complexes [20]. Under standard cell culture conditions, fiber binding lasted for at least 12 hr (Figure S2). To verify the specificity of fiber binding, we demonstrated that fiber molecules from the supernatants of Ad5-CRAD infected A549 cell cultures bound to CHO cells stably transfected with CAR cDNA, but not to the mock transfectants (Figure 3D). Furthermore, recombinant Ad5 fiber knob molecules, but not Ad35 fiber knob molecules, inhibited the binding of supernatant fiber molecules from Ad5-CRAD infected cultures to fresh A549 cells. Conversely, Ad35 fiber knob molecules, but not Ad5 fiber knob molecules, strongly inhibited the binding of supernatant fiber from Ad5F35-CRAD infected cultures to A549 cells (Figure 3E).

**Figure 4:**
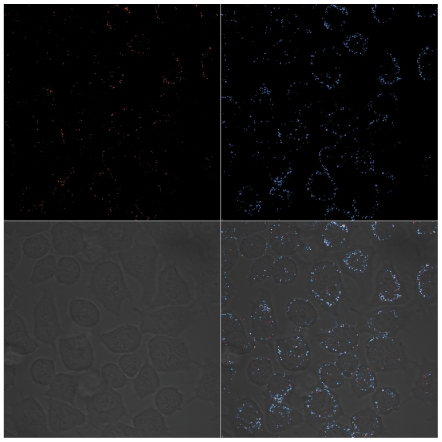
CLSM analysis of cell surface CAR expression and fiber binding. A549 cultures at 6 days post infection with Ad5-CRAD at a MOI of 1.0 were co-stained for cell surface CAR expression with the polyclonal rabbit anti-CAR 72 antibody (upper left) and fiber binding with mAb 4D2 (upper right) and analyzed in CLSM. The merged images (lower right) demonstrate the co-localization (white) of CAR and fiber molecules on cell surface.

To clarify whether decreased CAR intensity as assessed with RmcB mAb was due to fiber mediated receptor masking or to a down-regulated CAR expression, we used polyclonal CAR 72 [21] antibodies in combination with CLSM analysis to detect and visualize the localization of CAR molecules in A549 cells at day 6 following Ad5-CRAD infection at a MOI of 1.0. A patchy distribution of cell surface CAR staining as shown in Figure 2E was also detected with CAR 72 in non-infected cultures. In Ad5-CRAD infected cultures (with ∼1% GFP^+^ cells), the same CAR localization pattern as in the non-infected cultures was detected (Figure 4, upper left panel). Cell surface fiber binding was detected in a great majority of the cells, irrespective of the level of GFP or hexon expression (Figures 4, upper right panel and Figure S3). Importantly, vast majority of CAR and fiber labeling was co-localized as shown in the merged staining patterns (Figure 4, lower right panel and Figure S4). CAR and fiber internalization from cell surface were insignificant, as also shown in studies when A549 cells were incubated at 37°C with the supernatants of A549 cell cultures previously infected with Ad5-CRAD (Figure S5). Collectively, these findings demonstrate that fiber molecules produced from the few initially infected cells mask CAR on both CRAD infected and non-infected bystander cells.

### Mechanisms of Fiber Overproduction during Adenovirus Life Cycle

**Figure 5:**
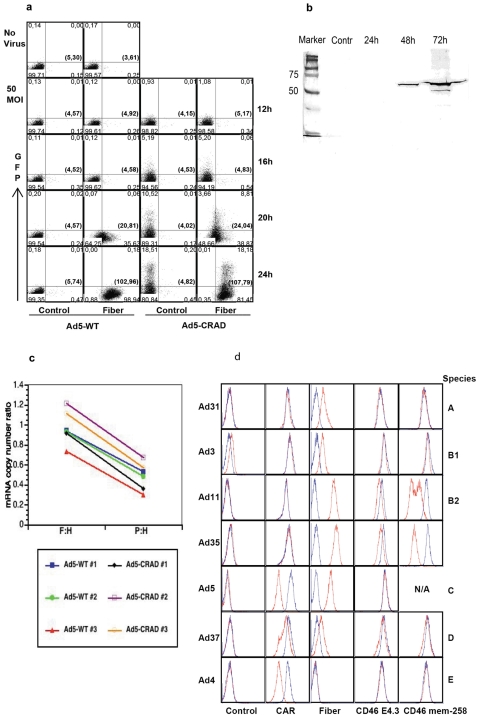
Mechanisms of fiber production during adenovirus life cycle. (A) Fiber secretion prior to adenoviral particle release. A549 cells were infected with WT Ad5 or Ad5-CRAD at a MOI of 50, cell surface fiber binding and GFP expression were assessed at the indicated time points post infection. Data are representative for A549 cell infection experiments. (B) Release of fiber upon cell lysis. A549 cells were infected with WT Ad5 at a MOI of 10 in DMEM containing 2% FCS. Forty µg of supernatant proteins harvested from the control non-infected culture, or from infected cultures at 24, 48 and 72 hr post infection were analyzed in Western-blot with 4D2 anti fiber mAb. (C) Abundance of fiber mRNA in Ad5 life cycle. Total RNA was extracted from A549 cells at 20 hr post infection with WT Ad5 or Ad5-CRAD as in (A), and analyzed for fiber, penton and hexon mRNA copy numbers in real-time RT-PCR. The paired ratios of fiber to hexon (F:H) and penton to hexon (P:H) mRNA copy numbers from each infection are depicted. (D) Fiber overproduction and receptor masking of various adenovirus serotypes. A549 cells were infected with indicated WT adenoviruses at 500 (Ad31, Ad3, and Ad37), 50 (Ad4), or 10 (Ad11, Ad35 and Ad5) viral particles per cell. Cell surface fiber binding and receptor intensity were analyzed upon visible CPE or day 10 post infection. In each histogram, the red and blue lines represent staining with infected and non-infected control cultures, respectively.

Next, we characterized the kinetics of fiber binding to CAR during WT Ad5 or Ad5-CRAD life cycle. A549 and 293 cells were super-infected with WT Ad5 or Ad5-CRAD at a MOI of 5 or 50. Cell surface fiber binding was assessed at 12, 16, 20 and 24 hr post infection. The kinetics of fiber release was comparable between WT Ad5 and Ad5-CRAD infected cells (Figure 5A). Analogous results were obtained also with 293 cells. Cell surface fiber binding was readily detected 20 hr after infection with both WT Ad5 and Ad5-CRAD at a MOI of 5. At 24 hr post infection, a high density of fiber binding was detected on all cells. Concomitantly, a 5-fold diminished CAR intensity as assessed by RmcB mAb was detected (data not shown). Similar fiber intensities were detected on GFP^+^ and GFP^−^ cells, suggesting that fibers secreted from cells with replicating virus spread to cells lacking the replicating virus. At 20 hr post infection, the vast majority of the viral particles were still intracellular (ratio to extracellular virus particles was 10^4^∶1). Thus, much adenoviral fiber protein is secreted prior to viral particle release. Further, the supernatants of A549 cells following super-infection with WT Ad5 were collected at 24, 48 and 72 hr post infection and assessed for the content of fiber molecules following depletion of cells. Increasing intensity of the monomer fiber band at ∼50 000 Da was detected in supernatants harvested at 48 and 72 hr post infection (Figure 5B), suggesting that large amounts of fiber molecules are also released after the infected cells are killed.

To further characterize the molecular mechanisms of fiber over-production, we used real-time RT-PCR to quantify the mRNA transcripts encoding adenoviral structural proteins at 20 hr post infection. With the hexon mRNA copy numbers as reference, the fiber to hexon and penton to hexon ratios were 0.97±0.17 and 0.49±0.14, respectively (N = 6, p<0.00001, paired *t* test) (Figure 5C). Thus, extremely significantly more fiber mRNAs were produced compared to penton base mRNAs. These data suggest that fiber over-production, and the relative ratio between fiber and penton base overproduction [15], appear to be a direct consequence of the transcript abundance.

We further investigated whether fiber mediated receptor masking is a general mechanism in adenovirus infection. The 51 adenovirus serotypes are divided into species A to F. While serotypes of species A, C, D, E and F bind CAR as a receptor [22], most B species adenoviruses bind CD46 [23]–[25]. High intensities of cell surface fiber binding were detected in A549 cells following CD46-binding species B Ad11 and Ad35 infection (Figure 5D). To these fiber bound cells, we used three different clones of anti-CD46 mAb, E4.3, J4.48 and also the CD46 blocking clone MEM-258 [25], to detect CD46 intensity. The binding of E4.3 and J4.48 clone was comparably diminished (J4.48 binding data not shown). However, a binding capacity at 10 to 20% of the control non-infected cells showed that MEM-258 binding was inhibited most in Ad11 and Ad35 fiber bound cells (Figure 5D). Since a down-regulated CD46 expression would have reduced the binding capacity of all 3 anti-CD46 mAbs to the same extent, these findings strongly suggest that CD46 is masked during infection with Ad11 and Ad35. In addition, we also studied the eventual receptor masking following infection of A549 cells with Ad31 (species A), Ad3 and Ad7 (species B), Ad37 (species D) and Ad4 (species E). Ad41 (species F) was not included in the analysis because we failed to obtain successful replication in this fastidious serotype. Under our conditions, we detected significantly diminished CAR intensity following Ad4, Ad5 and Ad37 infection in A549 cells (Figure 5D). Cell surface fiber binding was not detected following Ad4 infection, suggesting that the 4D2 mAb may not recognize Ad4 fiber molecules. Consistent with previous observations that Ad37 fiber can bind to both CAR and CD46 [26], [27], we now found that both CAR and CD46 were masked following Ad37 infection, but not to same extent as when infected with Ad11 and Ad35 (CD46) or Ad4 and Ad5 (CAR), which is in agreement with our previous report of Ad37 using sialic acid as a receptor [28]. However, measurable decrease of CAR or CD46 intensity were not detected in A549 cells following infection with Ad31, Ad3 and Ad7, suggesting that these serotypes may use either other molecules as main receptors, or not produce excessive amounts of fiber molecules, or both. Together, these data suggest that fiber mediated receptor masking is a shared mechanism during infection of CAR- or CD46-binding adenovirus serotypes.

### Effect of Fiber Overproduction and Its Secretion on CRAD Functionality

**Figure 6:**
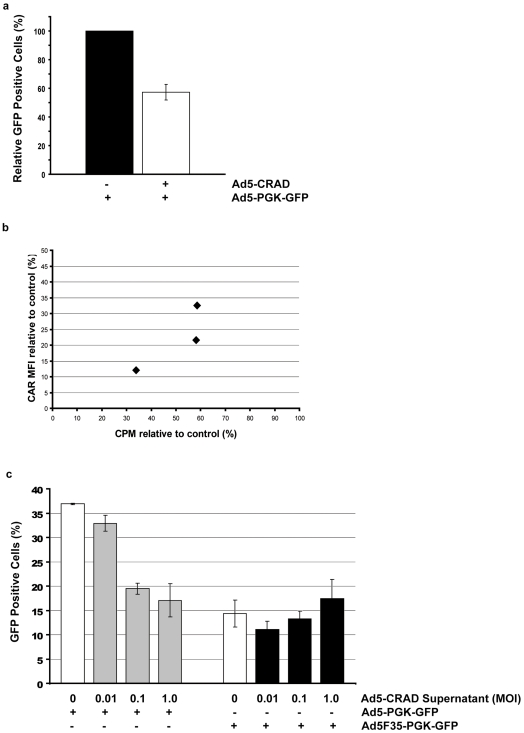
Consequences of fiber mediated receptor masking for CRAD infection. (A) Infectivity of receptor masked A549 cells by adenoviral vectors. A549 culture was first infected with Ad5-CRAD at a MOI 1.0. Six days later, cells were further infected with Ad5-PGK-GFP at a MOI of 10 and GFP expression was assessed 24 hr later. Data shown are the relative mean ± SD (n = 3) of the percentages of GFP^+^ cells in cultures infected by both Ad5-CRAD and Ad5-PGK-GFP compared to control cultures infected by Ad5-PGK-GFP only. (B) Adenovirus binding capacity to receptor masked cells. CAR intensity and ^35^S-Ad5 binding capacity to cells of A549 culture at day 6 post Ad5-CRAD infection (MOI  = 1.0) relative to cells of non-infected control culture of three independent experiments are shown. (C) Supernatants from Ad5-CRAD infected A549 cells conferred tropism-specific low infectivity to fresh A549 cells. A549 cell cultures were first incubated with supernatants of Ad5-CRAD infected cultures as in Figure 3B. Following extensive washing, cells were further infected with Ad5-PGK-GFP or Ad5F35-PGK-GFP at a MOI of 10. GFP expression was measured 24 hr later. The mean ± SD (n = 3) of the percentages of GFP^+^ cells are shown. The percentages of GFP^+^ cells in cultures incubated with the indicated Ad5-CRAD infection supernatant alone were deducted from the values presented in the grey or black bars.

To investigate the effect of fiber mediated receptor masking on efficiency of CRAD infection, A549 cells were first infected with Ad5-CRAD at a MOI of 1.0. Six days later, cells were further super-infected with replication defective Ad5-PGK-GFP. The frequencies of GFP expressing cells were measured 24 hr later. Because Ad5-CRAD also encodes *GFP*, more GFP^+^ cells would be expected in cultures infected with Ad5-CRAD and Ad5-PGK-GFP compared to the parallel control cultures infected only with Ad5-PGK-GFP. In contrast, the percentages of GFP^+^ cells in Ad5-CRAD infected cultures were only 60% of the control cultures without prior Ad5-CRAD infection (Figure 6A). Concordantly, cells from day 6 Ad5-CRAD infected cultures showed a CAR intensity correlated decrease in their binding capacity to ^35^S labeled WT Ad5 (Figure 6B). These data demonstrate that CRAD binding to and infection of receptor masked cancer cells can be significantly diminished.

The supernatants of Ad5-CRAD infected cultures contained both free fiber molecules and progeny CRADs. To assess the net supernatant effect in CRAD infection, we first incubated A549 cells at 37°C for 2 hr with the supernatants from A549 cell cultures that had been previously infected with Ad5-CRAD. Subsequently, cells were super-infected with Ad5-PGK-GFP or Ad5F35-PGK-GFP at a MOI of 10. Compared to cells incubated with the control supernatants from non-infected cultures, the supernatants from Ad5-CRAD infected cultures conferred an up to 50% reduction of the infectivity of CAR-binding Ad5-PGK-GFP; but the infectivity of CD46-binding Ad5F35-PGK-GFP was not affected (Figure 6C). These data further substantiate the conclusion that fiber overproduction and its resulting receptor masking of bystander cells hinder efficient CRAD infection.

**Figure 7:**
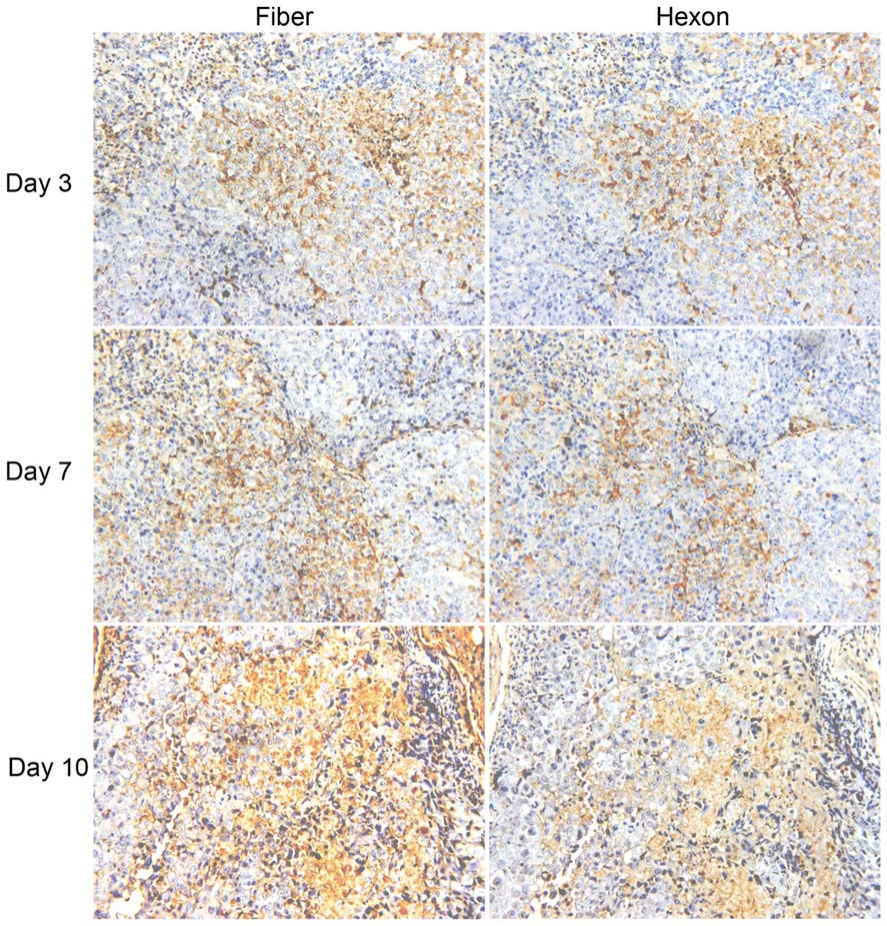
Extensive fiber production during CRAD treatment of xenograft tumors. Following a single intratumoral injection of 1×10^9^ PFU CNHK500, A549 cell xenograft tumors were dissected at day 3, 7 and 10 post injection and consecutive sections were stained for fiber and hexon proteins. Representative staining of fiber and hexon proteins are shown (×200).

To study whether the consequences of fiber overproduction are operational *in vivo*, a single intra-tumoral injection of 1×10^9^ plaque forming unit (PFU) *hTERT* promoter controlled CNHK500 CRAD [7] particles was performed with A549 cell xenografts in mice. Adenoviral hexon and fiber proteins were immunohistochemically localized in the tumors at day 3, 7 or 10 post injection. As shown by the localization of hexon expression, CRAD propagated within restricted regions in tumor (Figure 7). We consistently observed broader and more intense fiber staining compared to hexon staining in tumors at day 7 or 10 post CRAD injection. Similar findings of extensive fiber production were also observed in CNHK500 treatment of a liver carcinoma xenograft model (data not shown). These findings show that fiber molecules are dispersed from the regions with CRAD propagation, and strongly suggest that also *in vivo* fiber overproduction can limit CRAD progeny infection of bystander cells.

## Discussion

Our study reveals that during Ad5 based CRAD infection, large amounts of fiber molecules are secreted from cells prior to viral particle release. Fiber molecules produced from just 1 to 2% of cancer cells being CRAD infected are sufficient to mask adenoviral receptors in the bulk non-infected bystander cells. This receptor masking hinders CRAD progeny infectivity in bystander cells. We also reveal that fiber mediated receptor masking is a shared feature among CAR- or CD46-binding serotypes of adenoviruses. Our findings have fundamental implications for CRAD development and also provide a framework for understanding basic mechanisms underlying the natural course of adenovirus propagation.

**Figure 8:**
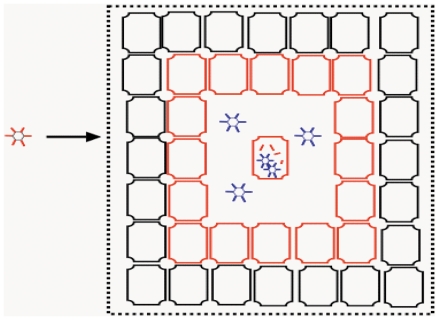
Effects of fiber overproduction and its resulting masking in CRAD cancer therapy and adenovirus propagation. The initially infected cells secrete large amounts of fiber molecules prior to progeny virus release. Consequently, the receptors on bystander cells are masked by fiber molecules (red). Receptor masked cells cannot be efficiently infected by progeny viruses (blue) when these are released upon cell lysis. This mechanism inhibits CRAD cell killing effects, but promotes a prolonged adenovirus host co-existence.

Although CRAD can be fiber re-targeted to more efficiently target and infect different types of cancer cells, and CRAD replication can be stringently engineered in a cancer cell-specific manner, the capacity of CRAD mediated cancer cell killing has been limited. This is reflected in a large body of studies which show that up to 1×10^9^ and 1×10^12^ viral particles are frequently applied in xenograft cancer therapy models and clinical trials, respectively [4], [6], [7]. CRAD cancer therapy in xenograft models was shown to inhibit cancer growth, but did not eradicate cancer [10], [11], and only marginal cancer therapy effects were observed in clinical trials [4]. The CRAD cancer cell killing capacity is dependent not only on the efficiency of CRAD replication, but also on the efficiency of progeny CRAD dispersal and propagation of infection within cancer tissue. However, the very first round of CRAD replication in a small fraction of cancer cells can already result in adenovirus receptor masking in the not yet infected bystander cancer cells. Consequently, receptor masked cancer cells cannot be efficiently infected by progeny CRADs when these are eventually released upon cell lysis. In the course of multiple rounds of CRAD infection, non-infected cancer cells proliferate and can also become receptor masked. Receptor masked cancer cells, when not infected by CRAD, may also proliferate (Figure 8). Due to higher binding avidity (combined strength of multiple bond interactions) between progeny viral particles and receptors compared to the binding between free fiber molecules and receptors, receptor masked bystander cells are bound and infected by CRAD progenies at low efficiency. The net therapeutic effect of CRAD will be dependent on the outcome between cancer cell proliferation and CRAD mediated cell killing. For CRAD to be efficient in cancer cell killing, fiber mediated receptor masking needs to be counteracted. In addition to overcoming the hurdle of neutralizing immunity [12], [13], it can be envisaged that non-Ad5 based CRADs, or Ad5 based CRADs engineered to express and secrete less fiber molecules would propagate more efficiently and have a more potent oncolytic capacity.

Fiber molecules associated with the adenovirus particle have been demonstrated to be important for adenovirus particle maturation, particle attachment to host cell, and the choice of intracellular trafficking routes of internalized particles [15], [29]. In the life cycle of several adenovirus serotypes, fiber and to a less extent penton base molecules are overproduced [14]–[16]. Penton base as well as penton can form dodecahedra [20]. The function of the free fiber molecules and dodecahedra has been elusive. Previous studies have demonstrated a role for Ad5 fiber molecules in disrupting CAR-mediated inter-epithelial cell-cell adhesion which could facilitate adenovirus spread in the course of a natural infection process [17]. However, our study strongly suggests that the functional consequence of fiber overproduction is unlikely to be restricted to disrupting CAR-mediated cell-cell adhesion [17]. Besides CAR binding Ad5, Ad4 and Ad37, fiber mediated receptor masking was also detected during infection with CD46 binding Ad11 and Ad35 under our conditions. Unlike CAR, CD46 does not form inter-epithelial cell homodimers that contributes to upholding tight junctions and epithelial integrity. Although it is possible that some adenovirus serotypes (e.g. Ad3 and Ad7) utilize cell surface receptors other than CAR and CD46 [25], [30], and the possibility also exists that the antibodies used in this study did not detect the fiber molecules of the remaining serotypes, or these fiber molecules bind to CAR with a lower affinity, our data strongly suggest that fiber mediated receptor masking in bystander cells is a conserved, fundamental mechanism for the adenovirus-host cell interaction, which is likely a critical modulator of adenovirus propagation.

There are significant differences in the pathogenicity and course of disease among different serotypes, and infection of certain serotypes can persist for years in immune competent individuals [1], [4], [31]. Host cells when infected with adenovirus, activate host-antiviral defense mechanisms, such as the production of interferon molecules and inflammatory cytokines [32]. Adenoviruses on the other hand are endowed with counter-offense capacities against such cellular antiviral defense. E1A and E1B products for example, can suppress the transcription of interferon-stimulated genes, and E3 19 k proteins can inhibit cell-surface expression of HLA class I molecules [33]. These counter-offense mechanisms may protect adenovirus-infected cells from attack by innate and adaptive immune responses and thereby contributes to adenovirus persistency. However, these virus host cell interaction mechanisms are restricted to the survival of adenovirus-infected cells. Using CRAD also encoding for GFP, we can distinguish cells with adenovirus replication from those without, and assess what consequence a small population of infected cells can have on the surrounding non-infected cells. Our data have demonstrated that adenovirus infection in a small fraction of cells can result in receptor masking in the bulk of non-infected bystander cells (illustrated in Figure 8). Fiber mediated receptor masking provides a relative “protection” from progeny virus infection to the non-infected bystander cells. This can be advantageous to adenovirus propagation and persistency as compared to immediate killing of all host cells following infection. Instead, adenoviruses benefit from a co-existence between virus and host cells, with a lower rate of viral production compared to the acute infection phase. In this context, we hypothesize that the extent of fiber overproduction and secretion could serve as a virulence factor for different adenoviruses. In agreement with this hypothesis, persistent clinical infections have been observed by Ad4, Ad5, Ad11 and Ad35 [1], [31], [34], [35], the serotypes demonstrated to be capable of causing fiber overproduction and receptor masking in this study.

Our findings of excessive production of viral attachment proteins such as fiber in the few initially infected cells, and the resulting receptor masking in the bulk of non-infected bystander cells are unlikely unique to adenoviruses. Excessive production of defective viral particles and attachment proteins are common during natural infection with various types of viruses. It can be envisaged that attachment protein overproduction in infected cells and the resulting viral receptor modulation in non-infected bystander cells is a frequent phenomenon during viral infection. In fact, adenovirus receptors CAR, sialic acids and CD46 are also utilized as host cell receptors for other types of viruses. For example, CD46 is utilized as a receptor by vaccine strains of measles virus, human herpes virus type 6 (HHV-6) and bovine viral diarrhoea virus [36], and sialic acid is a receptor for influenza viruses [37]. As an integral member of tight junction, CAR is structurally and functionally similar to other cell adhesion receptors, such as CD155 for polioviruses, junction adhesion molecule for reoviruses, and nectins for herpes simplex viruses [38]. Interestingly, the reovirus attachment protein σ1 is structurally similar to adenovirus fiber molecules [39]. And, σ1 proteins from reovirus type 1 and type 3 determine the pattern of reovirus spread from the initial infection site to the central nervous system [40]. Although the eventual overproduction of attachment protein in infected cells and the resulting viral receptor modulation in non-infected bystander cells remain to be investigated for these viruses, receptor down-modulation or masking may inhibit superinfection of host cells by progeny virus, or facilitate virus escape from infection site to the secondary organs or tissues, or enhance cell sensitivity to antibody mediated cell killing. Thus, overproduction of viral attachment molecules and its resulting modulation of viral receptors may contribute to control the natural infection course of various viruses.

In summary, our studies demonstrate a fundamentally detrimental role of fiber overproduction in CRAD functionality, and strongly suggest fiber overproduction and its resulting receptor masking as a key factor contributing to adenovirus host co-existence. These findings have fundamental implications for the development of antiviral strategies and human gene therapy.

## Materials and Methods

### Viruses

Ad5-CRAD and Ad5-PGK-GFP have been previous described [6], [9]. In the Ad5-CRAD, the E1 sequences from Ad5 nucleotides 458 to 3533 are engineered downstream of the hTERT promoter, and the GFP cDNA is engineered downstream of E1B using the internal ribosomal entry site sequence [6]. The Ad5F35-CRAD was generated using a modified AdEasy system [9]. Briefly, an E1A encoding expression cassette controlled by *hTERT* promoter and rabbit β-globin intron 2 and polyA sequences was engineered into pShuttle, and inserted into pAdEasy1/F35 via homologous recombination in *E coli* BJ5183 strain [5], [9]. The E3 region is deleted in both Ad5-CRAD and Ad5F35-CRAD. Viruses were rescued in 293 cells according to standard procedures [9]. WT viruses were expanded in A549 cells. All recombinant viruses and WT viruses were purified with the two-step CsCl centrifugation procedure. Physical particle titer was measured at OD260 [41] and the titer of infectious units (IU) was determined using Adeno-X rapid titer kit (Clontech). The viral preparations have a physical particle to IU ratio of 10∶1. MOIs are in IUs/cell.

### Cell Culture and Adenovirus Infection

CHO-CAR and CHO-control cells were cultured with MEM-alpha medium-nucleotides supplemented with 5% heat inactivated dialyzed fetal calf serum (FCS), 100 U/ml penicillin, and 100 µg/ml streptomycin. A549, HT1080, LoVo, SW480 and SKBr3 cells were cultured in DMEM supplemented with 10% heat inactivated FCS, 1% non-essential amino acids, 100 U/ml penicillin, and 100 µg/ml streptomycin. Cells were seeded at 12 000 cells/well in 12-well plates and infected with CRAD after 2 hr of culture. To assess cell killing, living cells in each well determined by trypan blue exclusion were counted at day 7 post infection.

### Flow Cytometry Analysis

Fiber binding and receptor intensity in CRAD or WT Ad infected cultures were assessed at the indicated days post infection, or at the appearance of CPE, or latest at day 10 post adenovirus infection. Antibody staining procedures were performed on ice. Cells were first incubated with either anti-CAR or anti-CD46, or isotype-matched control mAb for 15 min. Following PBS washing, cells were incubated with Alexa Fluor® 647 conjugated goat anti-mouse IgG antibody (Molecular Probes) for 15 min. Finally, cells were washed and resuspened in PBS containing 1 µg/ml 7-aminoactinomycin D (7-AAD, Sigma). Living cells negatively stained with 7-AAD were analyzed. We used clone RmcB (Upstate) and E1-1 (Abcam) anti-CAR mAbs; clone E4.3 (BD), MEM-258 (BioLegend), and J4.48 (Chemicon) anti-CD46 mAbs. The anti fiber mAbs (clone 4D2 and 2D6) were from GeneTex. MOPC-21 (IgG1, Sigma) and MG2a00 (IgG2a, CALTAG Laboratories) served as isotype controls.

### Preparation of Free Fiber Containing Supernatant and Endogenous Fiber Blocking Assay

Supernatants were harvested from control or Ad5-CRAD infected A549 cultures at day 7 post infection, filtered through a 0.22 µM sterile filter (Sarstedt). When free fiber containing supernatants were used, supernatants were first spun twice at 108 000 g for 1 hr, filtered through a Jumbosep^TM^ centrifuge device with a 300 kDa cut-off (Pall Corporation) and followed by the 0.22 µM sterile filter. A549 cells, CHO-CAR and CHO-control transfectants were seeded in 24-well plates at 10^5^ cells/well. After 24 hr of culture, 0.5 mL supernatants from control or Ad5-CRAD infected A549 cultures were added and cells were incubated for 2 hr at 37°C. Following twice PBS washing, cells were either directly analyzed in flow cytometry for fiber binding and receptor intensity, or infected with Ad5-PGK-GFP at a MOI of 10. GFP expression was analyzed 24 hr later.

In some experiments, seeded A549 cells were first incubated for 2 hr at 37°C with 2 µg/ml recombinant Ad5 or Ad35 fiber knob molecules [42]. Following 2x PBS washing, 0.5 mL supernatant from Ad5-CRAD or Ad5F35-CRAD infected cultures were added, and cells were further incubated for 2 hr at 37°C. Finally, cell surface binding of endogenous fiber, but not the knob molecules, was detected with 4D2 mAb in flow cytometry.

### Western-Blot Analysis

A549 cells were infected with WT Ad5 at a MOI of 10 in DMEM containing 2% FCS. Supernatants were harvested at 24, 48 and 72 hr post infection. Following depletion of cells at 4 000 g for 10 min, a cocktail of protease inhibitors (Sigma, p-2714) was added to one tenth of the supernatant volume. Forty µg of supernatant proteins were separated with SDS-PAGE and transferred to PVDF membrane. Following blocking with 5% BSA and 0.5% goat serum, the membrane was incubated with 1.0 µg/ml 4D2 anti fiber mAb at 4°C overnight. Subsequently, the membrane was incubated with goat anti-mouse IgG-HPR and visualized with Opti-4CNTM substrate kit.

### Adenovirus Binding and Re-Infection Assay in CRAD Infected Cultures

A549 cells were infected with Ad5-CRAD at a MOI of 1.0. On day six post infection, cells were trypsinized and either analyzed for cell surface CAR intensity using flow cytometry, or assessed in virus binding assay as described below. Following twice washing in binding buffer (DMEM, 2 mM MgCl_2_, 1% BSA and 20 mM HEPES), cells were resuspended at 2×10^5^ cells/100 µL and incubated for 20 min on ice. S^35^ labelled WT Ad5 viruses were then added. Following incubation for 1 hr on ice and 2x washing in binding buffer, cells were pelleted and resuspended in scintillation fluid. The cell-associated radioactivity was counted with a liquid scintrillation analyzer (Tri-Carb 2800 TR, Perkin Elmer). The day 6 Ad5-CRAD infected or uninfected control A549 cultures were re-infected with Ad5-PGK-GFP at a MOI of 10 and analyzed for GFP expression 24 hr later using flow cytometry.

### CLSM Analysis

A549 cell suspension (10^4^ cells/mL) was mixed with dilutions of Ad5-CRAD at a MOI of 1. The cell/virus mixture was plated either 0.5 mL/well in 12-well plates or 0.25 mL/well in 8-well µ-slide (IBIDI) and incubated at 37°C. At day 6 post infection, cultures in 12-well plates were analyzed by flow cytometry for GFP expression, cell surface fiber binding and CAR intensity. The parallel cultures in µ-slides were prepared for CLSM analysis. Following washing with PBS, cells were incubated in 5% goat serum for 20 min, followed by incubation with RmcB mAb (1∶200), or with rabbit polyclonal CAR 72 antibodies (1∶500), or rabbit polyclonal anti Ad5 hexon antibodies (1∶1000, Abcam) in combination with anti fiber 4D2 mAb (1∶200) for 90 min. The slides were then washed and incubated for 30 min with 1∶400 diluted goat anti-mouse Alexa Fluor® 647, or combined with goat anti-rabbit Alexa Fluor® 568 (Molecular Probes). For antibody specificity control and adjustment of auto-fluorescence, staining excluding the primary antibodies was included in every experiment. After rinsing, DAPI (0.1 µM) was added to all wells. All staining procedures were performed on ice. GFP expression, CAR and fiber localization were assessed with a Zeiss LSM 510 Meta microscope.

In experiments where A549 cultures were incubated with supernatants from A549 cultures previously infected with Ad5-CRAD or directly infected with Ad5-CRAD as described above, cells were fixed (4% paraformaldehyde for 30 min) and permeabilized (0.3% Triton x-100 for 5 min), and subsequently stained with CAR 72 or anti-hexon and 4D2 antibodies and analyzed in CSLM as above.

### Kinetics of Fiber Secretion and Real-Time RT-PCR

A549 or 293 cells were seeded at 10^5^ cells/well in 24-well plates. Twenty four hr later, cells were infected with Ad5-CRAD or WT Ad5 at a MOI of 5 or 50. Cell surface fiber binding was analyzed by flow cytometry at 12, 16, 20 and 24 hr post infection. At 20 hr post infection, supernatants were saved, cells were detached from the plate and resuspended in the same volume as the supernatant. The harvested supernatant and cell fractions were 3x frozen/thawed, filtered through 0.22 µM sterile filter, and titered in a standard plaque assay. Cells at 20 hr post infection with Ad5-CRAD or WT Ad5 as above were also harvested for total RNA extraction. Total RNA was reverse transcribed and used for real-time PCR assessment of hexon, penton and fiber mRNA abundance as detailed in Data S1.

### Xenograft Experiment and Immunohistochemistry Analysis

This study was approved by the ethical committee of Shanghai Eastern Hepatobiliary Surgical Hospital, and we followed the criterion in animal experiments established by American Veterinary Medical Association to dispose animals scientifically and humanely [43]. Four weeks old BABL/c-Nude mice were injected subcutaneously with 1×10^7^ A549 cells in 100 µL PBS. Three weeks later, tumors with a diameter of about 5 mm were injected with 100 µL Ad buffer (10 mmol/L Tris-HCl pH 8.0, 2 mmol/L MgCl_2_, and 4% sucrose) containing 1×10^9^ PFU of CNHK500 [7] or Ad buffer alone. Five animals were killed on 3, 7, or 10 days post CRAD injection. Tumors were dissected, fixed in formalin and embedded in paraffin. Consecutive sections of 5 µm were prepared and stained with 1∶200 diluted mouse anti-hexon antibody (MAB8052, Chemicon) or anti-fiber 4D2 mAb at 4°C overnight. Subsequently, sections were stained with biotin-conjugated goat-anti-mouse antibody and streptavdin-peroxidase reagents (Maxim Laboratories, Inc), each for 30 min at 37°C. Following diaminobenzidine development and counterstaining with hematoxylin, the hexon and fiber staining patterns were evaluated under a magnification of × 200.

## Supporting Information

Data S1 (0.03 MB DOC)

Figure S1 Cell surface fiber binding and decrease of CAR intensity in cancer cell lines following Ad5-CRAD infection. Concomitant cell surface fiber binding and decrease of CAR intensity in both infected and non-infected bystander cells at 7 days post infection with Ad5-CRAD are shown. Data are representative for 3 independent experiments performed with each cell line.
(2.16 MB TIF)

Figure S2 Sustained binding of fiber or recombinant knob molecules to receptors. (A) A549 cells were incubated for 2 hr at 37°C with <300 KDa supernatant from cultures previous infected with Ad5-CRAD. Cells were extensively washed, further cultured for indicated hr at 37°C, and assessed for cell surface fiber binding and CAR intensity by flow cytometry. The mean and SD (n = 3) of fiber binding MFI and relative CAR intensity are shown. (B) The mean and SD (n = 3) of CAR or CD46 staining intensity relative to control cells in A549 cells at 48 hr following a 2 hr incubation with knob molecules are shown.
(0.50 MB TIF)

Figure S3 CLSM analysis of hexon and fiber on A549 cell culture previously infected with Ad5-CRAD at low MOI. Following fixation with paraformaldehyde and permeabilization with Triton x-100, cells were co-stained with anti-hexon and anti-fiber antibodies, and subsequently with their corresponding secondary antibodies. Nuclear staining was performed with DAPI (upper left). Representative staining of hexon (upper right), GPF (middle left), and fiber (middle right) show hexon and GFP only in the infected cell but fiber on the surface of a great majority of the cells. Merged image is shown in the lower left panel.
(3.54 MB TIF)

Figure S4 CLSM analysis of cell surface fiber binding and CAR distribution in A549 cell cultures infected with Ad5-CRAD. Representative staining patterns of CAR (upper left), fiber (upper right), GFP (lower left) in the Ad5-CRAD infected A549 cultures with ∼2% GFP+ cells are shown. The merged image (white, lower right) shows cell surface co-localization of CAR and fiber molecules. Staining was performed in living cells on ice.
(0.75 MB TIF)

Figure S5 CLSM analysis of fiber binding and CAR distribution in A549 cells following incubation with supernatant of A549 culture previously infected with Ad5-CRAD. A549 cells were incubated with supernatant of A549 culture previously infected with Ad5-CRAD at 37°C for 2 hr. Following fixation with paraformaldehyde and permeabilization with Triton x-100, cells were co-stained with CAR 72 and 4D2 primary antibodies, and subsequently with their corresponding secondary antibodies. Represent staining patterns of CAR (upper left), fiber (upper right), and DAPI (lower left) are shown. The merged image (yellow, lower right) shows that most fiber molecules co-localized with CAR on the cell surface.
(4.22 MB TIF)
