# Supplementary material for: Fiber Mediated Receptor Masking in Non-Infected Bystander Cells Restricts Adenovirus Cell Killing Effect but Promotes Adenovirus Host Co-Existence
Source: PLoS One. 2009 Dec 29;4(12):e8484. doi: 10.1371/journal.pone.0008484 (PMC2793518; doi:10.1371/journal.pone.0008484)
Supplement: Data S1 — (0.03 MB DOC) [file pone.0008484.s001.doc]

**Supplemental data**

**Fiber mediated receptor masking in non-infected bystander cells restricts adenovirus cell killing effect but promotes adenovirus host co-existence**

Rebetz J. et al.

**Supplemental Experimental Procedures**

**Real-time RT-PCR**

A549 cells were seeded at 5 x 10^5^ cells per well in 6-well plates. After incubation for 24 hr at 37°C, cells were infected with Ad5-CRAD or WT Ad5 at a MOI of 10. Twenty hr later, cells were harvested for RT-PCR analysis of adenovirus structure gene expression. For analyzing CAR and CD46 expression, cells were seeded at 1.2 x 10^4^ cells per well in 6-well plates, infected with Ad5-CRAD at a MOI of 1, and harvested when 2-5% of the total cells were GFP^+^. Total RNA was extracted with an RNAEasy® mini kit (Qiagen) according to manufacturer’s instruction. The RNA preparations were treated with DNAse I. Briefly, 2 μg of total RNA were incubated for 30 min at 37°C with 10 U RNase Out (Invitrogen), 0.5 U DNaseI and buffer from a RNase free DNase set (Qiagen). Subsequently, 2 μL of 2 mM EDTA were added and the samples were further incubated for 10 min at 65°C. The RNA was then reverse-transcribed to cDNA with Superscript III first strand synthesis system (Invitrogen). The amount of mRNA was analyzed on a LightCycler 1.2 with a LightCycler® FastStart DNA Master^PLUS^ STBR Green I kit (Roche).

To estimate the copy numbers of hexon, penton and fiber mRNA molecules, a known concentration of pBHG10 [1] containing the three genes was used as the standard. For analyzing the CAR and CD46 mRNAs in infected and control cells, the relative expression level was calculated with the REST program [2] using GAPD and HPRT1 as reference genes.

Primers used in the analysis:

Penton forward: 5´-CAACAAGTCAACGGATGTGG-3’

Penton reverse: 5´-GTTCACATTTGGCATGTTGG-3´

Hexon forward: 5´-GGCCTACCCTGCTAACTTCC-3´

Hexon reverse: 5´-TGGCGTAGAGAAGGTTTTGG-3´

Fiber forward: 5´-ACTCACCATGCAATCACAGG-3´

Fiber reverse: 5´-GGGCTCTTTCAAGTCAATGC-3´

CAR forward: 5´-TAAGCCTTCAGGTGCGAGAT-3´

CAR reverse: 5´-CCATGAAGTGGGCATTTTCT-3´

CD46 forward: 5´-ACAACCTGGTTTGCCAGTTC-3´

CD46 reverse: 5´-GGAAAGCAGGCAATCTGTTC-3´

GAPD forward: 5´-CCCTGTTGCTGTAGCCAAATTC-3´

GAPD reverse: 5´-TCTCCTCTGACTTCAACAGCGAC-3´

HPRT1 forward: 5´-CAAGCTTGCTGGTGAAAAGGA-3´

HPRT1 reverse: 5´-ACTAAGCAGATGGCCACAGAA-3´

**Duration of the binding of recombinant fiber knob or endogenous Ad5 fiber molecules to receptors on A549 cells**

A549 cells seeded in 24-well plates were incubated for 2 hr at 37°C either with 2 µg/ml of recombinant Ad5 or Ad35 knob molecules, or with 1 ml of ≤ 300 KDa free fiber containing supernatant from A549 cultures previously infected with Ad5-CRAD. Cells were 2x washed with PBS and subsequently cultured at 37°C in DMEM containing 10% FCS. After different time intervals, cells were harvested, stained with 4D2 anti-fiber mAb, or RmcB anti-CAR mAb, or E4.3 anti-CD46 mAb, and analyzed by flow cytometry.

**Supplemental Reference**

1. Ng, P., et al., *An enhanced system for construction of adenoviral vectors by the two-plasmid rescue method.* Hum Gene Ther, 2000. **11**(5): p. 693-9.

2. Pfaffl, M.W., G.W. Horgan, and L. Dempfle, *Relative expression software tool (REST) for group-wise comparison and statistical analysis of relative expression results in real-time PCR.* Nucleic Acids Res, 2002. **30**(9): p. e36.
