# Supplementary figures and images for: Fiber Mediated Receptor Masking in Non-Infected Bystander Cells Restricts Adenovirus Cell Killing Effect but Promotes Adenovirus Host Co-Existence
Source: PLoS One. 2009 Dec 29;4(12):e8484. doi: 10.1371/journal.pone.0008484 (PMC2793518; doi:10.1371/journal.pone.0008484)

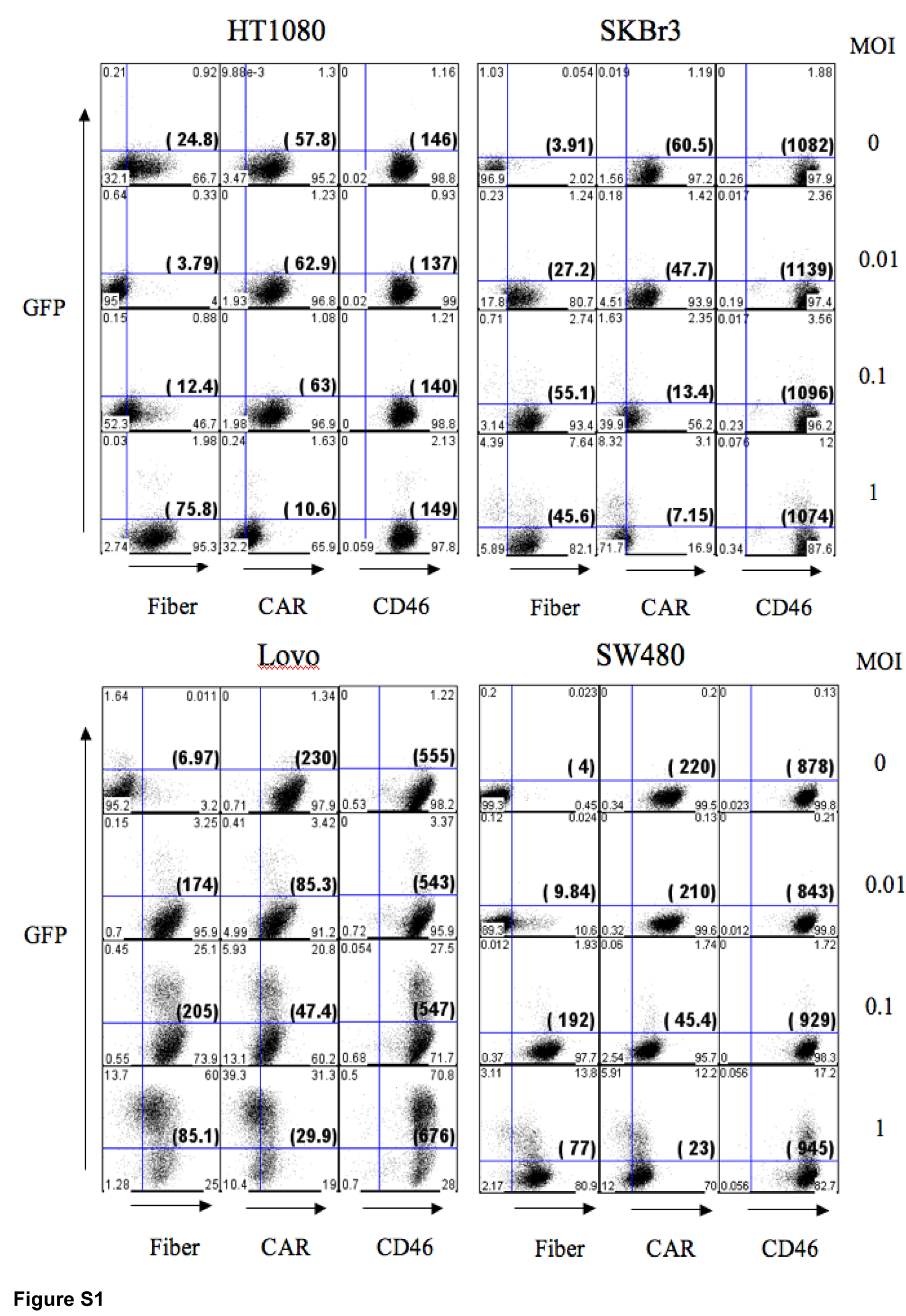

Supplement: Figure S1 — Cell surface fiber binding and decrease of CAR intensity in cancer cell lines following Ad5-CRAD infection. Concomitant cell surface fiber binding and decrease of CAR intensity in both infected and non-infected bystander cells at 7 days post infection with Ad5-CRAD are shown. Data are representative for 3 independent experiments performed with each cell line. (2.16 MB TIF) [file pone.0008484.s002.tif]

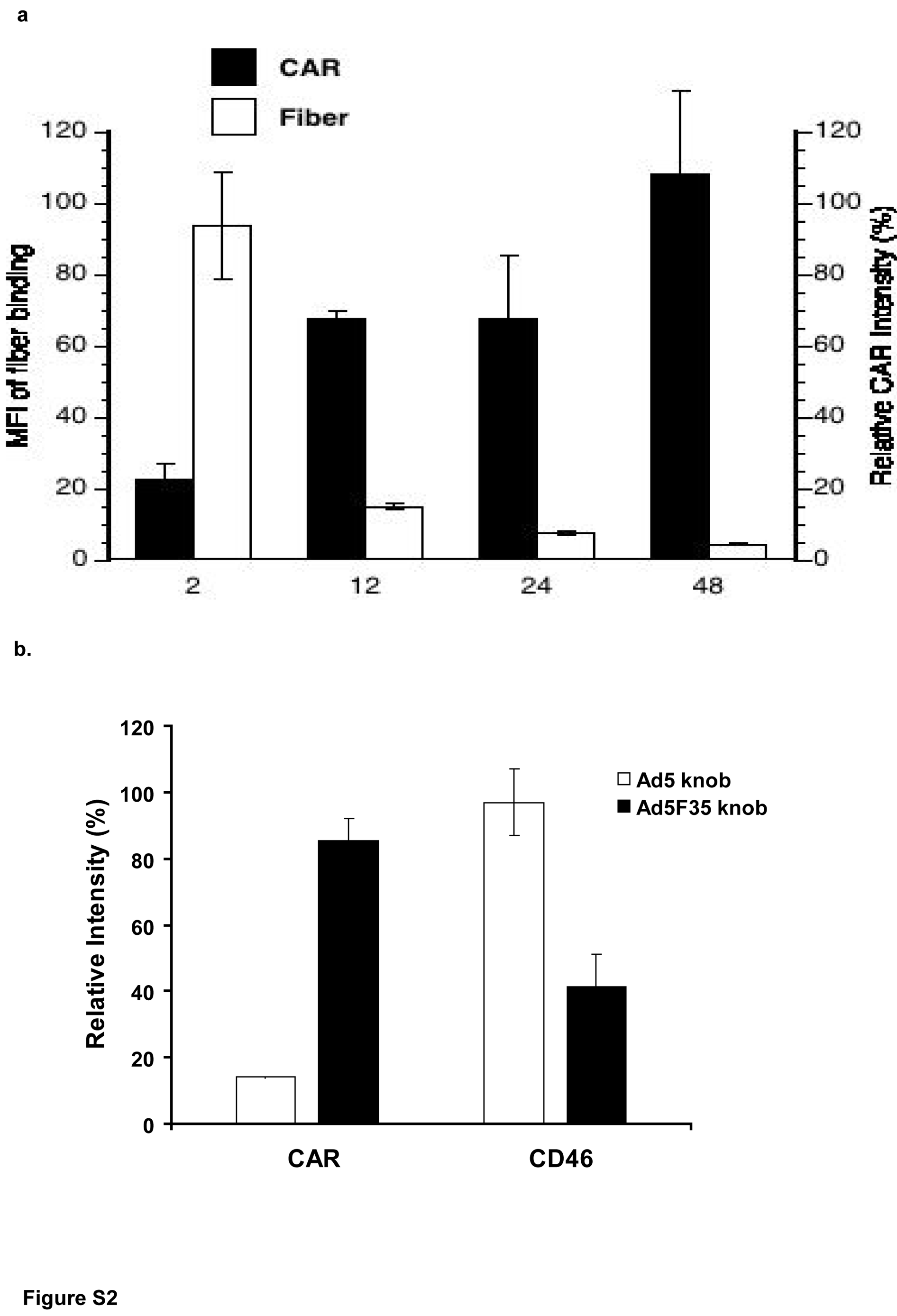

Supplement: Figure S2 — Sustained binding of fiber or recombinant knob molecules to receptors. (A) A549 cells were incubated for 2 hr at 37°C with <300 KDa supernatant from cultures previous infected with Ad5-CRAD. Cells were extensively washed, further cultured for indicated hr at 37°C, and assessed for cell surface fiber binding and CAR intensity by flow cytometry. The mean and SD (n = 3) of fiber binding MFI and relative CAR intensity are shown. (B) The mean and SD (n = 3) of CAR or CD46 staining intensity relative to control cells in A549 cells at 48 hr following a 2 hr incubation with knob molecules are shown. (0.50 MB TIF) [file pone.0008484.s003.tif]

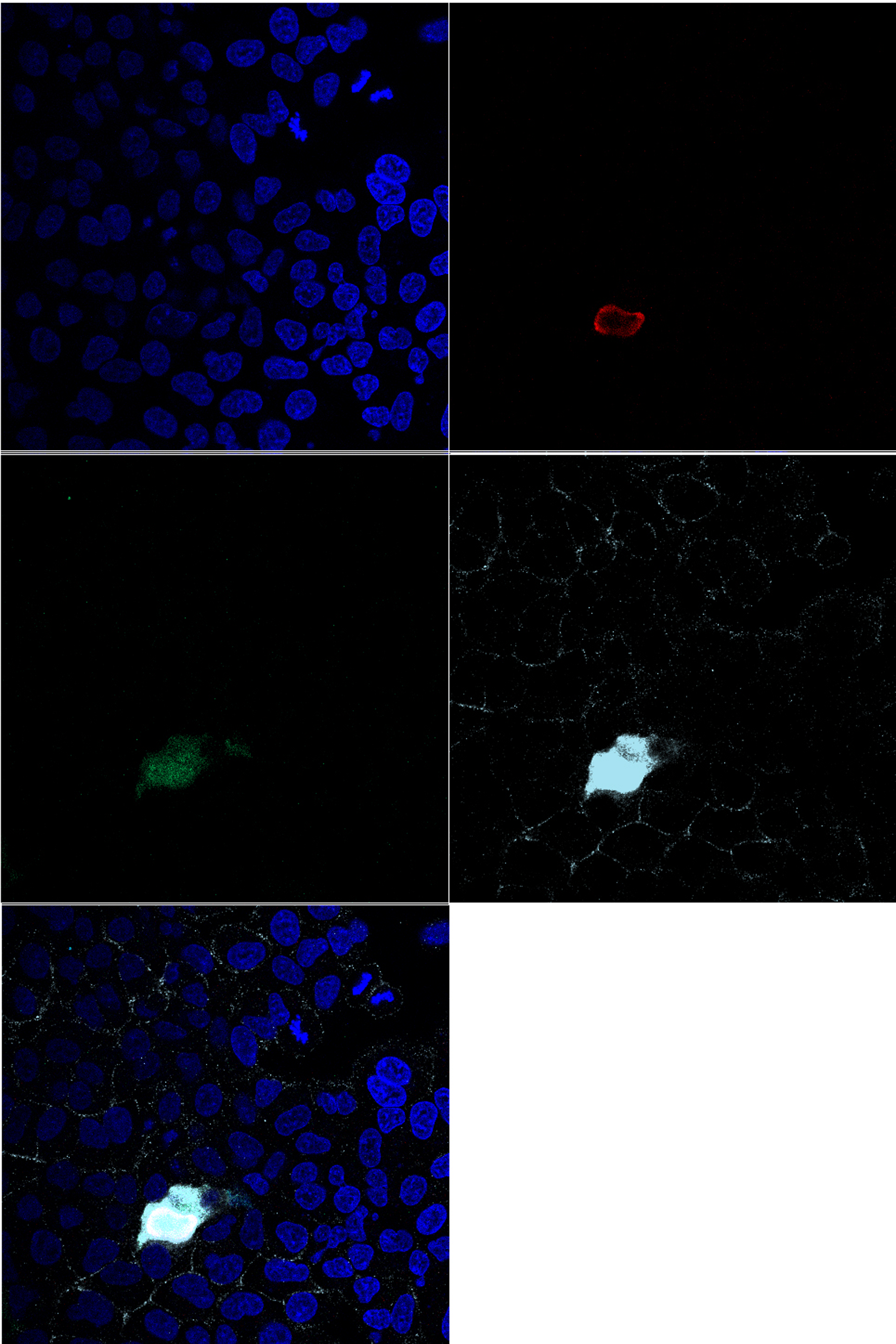

Supplement: Figure S3 — CLSM analysis of hexon and fiber on A549 cell culture previously infected with Ad5-CRAD at low MOI. Following fixation with paraformaldehyde and permeabilization with Triton x-100, cells were co-stained with anti-hexon and anti-fiber antibodies, and subsequently with their corresponding secondary antibodies. Nuclear staining was performed with DAPI (upper left). Representative staining of hexon (upper right), GPF (middle left), and fiber (middle right) show hexon and GFP only in the infected cell but fiber on the surface of a great majority of the cells. Merged image is shown in the lower left panel. (3.54 MB TIF) [file pone.0008484.s004.tif]

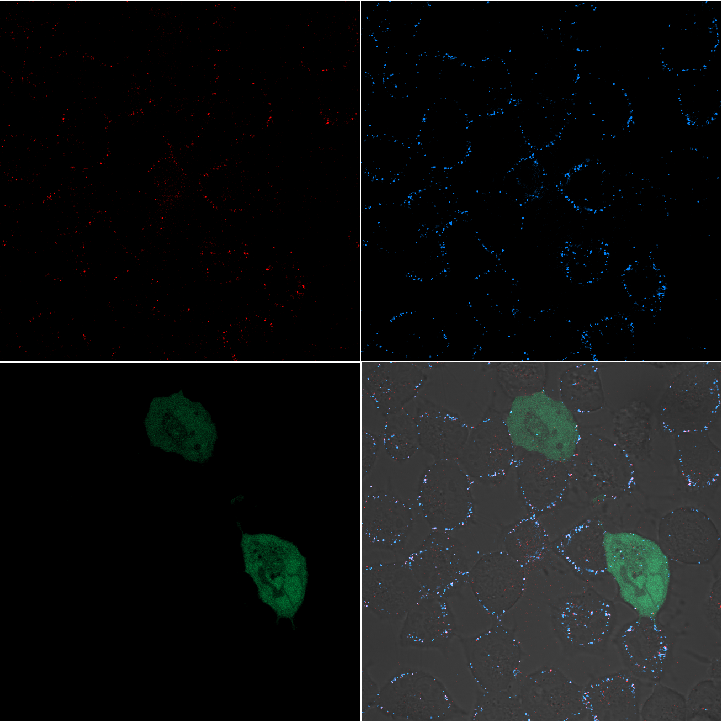

Supplement: Figure S4 — CLSM analysis of cell surface fiber binding and CAR distribution in A549 cell cultures infected with Ad5-CRAD. Representative staining patterns of CAR (upper left), fiber (upper right), GFP (lower left) in the Ad5-CRAD infected A549 cultures with ∼2% GFP+ cells are shown. The merged image (white, lower right) shows cell surface co-localization of CAR and fiber molecules. Staining was performed in living cells on ice. (0.75 MB TIF) [file pone.0008484.s005.tif]

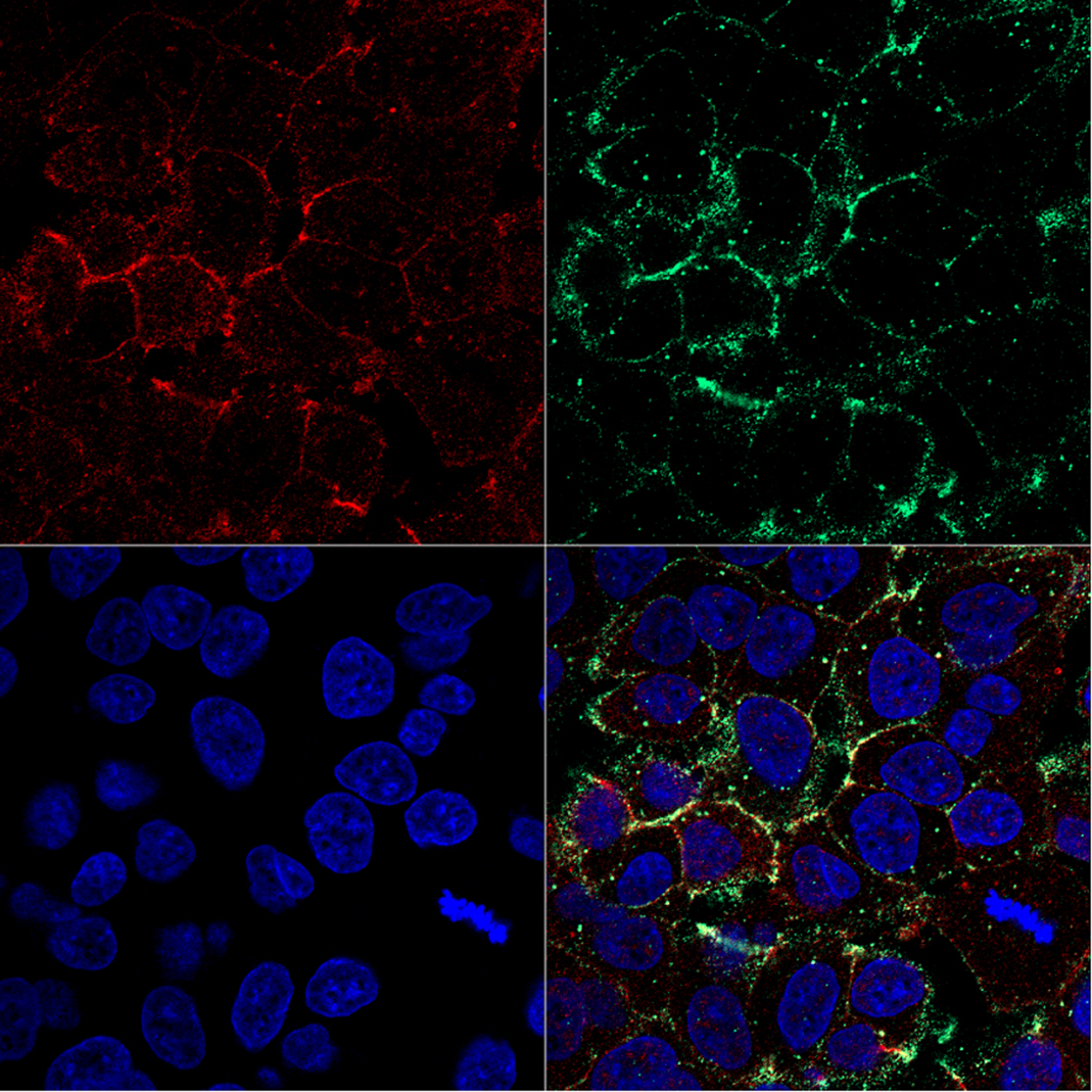

Supplement: Figure S5 — CLSM analysis of fiber binding and CAR distribution in A549 cells following incubation with supernatant of A549 culture previously infected with Ad5-CRAD. A549 cells were incubated with supernatant of A549 culture previously infected with Ad5-CRAD at 37°C for 2 hr. Following fixation with paraformaldehyde and permeabilization with Triton x-100, cells were co-stained with CAR 72 and 4D2 primary antibodies, and subsequently with their corresponding secondary antibodies. Represent staining patterns of CAR (upper left), fiber (upper right), and DAPI (lower left) are shown. The merged image (yellow, lower right) shows that most fiber molecules co-localized with CAR on the cell surface. (4.22 MB TIF) [file pone.0008484.s006.tif]
